# Long-Term Assessment of Air Quality and Identification of Aerosol Sources at Setúbal, Portugal

**DOI:** 10.3390/ijerph17155447

**Published:** 2020-07-28

**Authors:** Alexandra Viana Silva, Cristina M. Oliveira, Nuno Canha, Ana Isabel Miranda, Susana Marta Almeida

**Affiliations:** 1 Centro de Ciências e Tecnologias Nucleares (C2TN), Instituto Superior Técnico, Universidade de Lisboa, Estrada Nacional 10, Km 139.7, 2695-066 Bobadela LRS, Portugal; alexandra.silva@kevolution.org (A.V.S.); smarta@ctn.tecnico.ulisboa.pt (S.M.A.); 2 Centro de Química Estrutural (CQE), Faculdade de Ciências da Universidade de Lisboa, Campo Grande, 1749-016 Lisboa, Portugal; cmoliveira@fc.ul.pt; 3 Centro de Estudos do Ambiente e do Mar (CESAM), Departamento de Ambiente e Ordenamento, Universidade de Aveiro, 3810-193 Aveiro, Portugal; miranda@ua.pt

**Keywords:** air pollutants, particulate matter, monitoring, seasonality, chemical characterization, source apportionment

## Abstract

Understanding air pollution in urban areas is crucial to identify mitigation actions that may improve air quality and, consequently, minimize human exposure to air pollutants and their impact. This study aimed to assess the temporal evolution of the air quality in the city of Setúbal (Portugal) during a time period of 10 years (2003–2012), by evaluating seasonal trends of air pollutants (PM_10_, PM_2.5_, O_3_, NO, NO_2_ and NO_x_) measured in nine monitoring stations. In order to identify emission sources of particulate matter, PM_2.5_ and PM_2.5–10_ were characterized in two different areas (urban traffic and industrial) in winter and summer and, afterwards, source apportionment was performed by means of Positive Matrix Factorization. Overall, the air quality has been improving over the years with a decreasing trend of air pollutant concentration, with the exception of O_3_. Despite this improvement, levels of PM_10_, O_3_ and nitrogen oxides still do not fully comply with the requirements of European legislation, as well as with the guideline values of the World Health Organization (WHO). The main anthropogenic sources contributing to local PM levels were traffic, industry and wood burning, which should be addressed by specific mitigation measures in order to minimize their impact on the local air quality.

## 1. Introduction

Air pollution is considered one of the main environmental problems that countries face nowadays taking into account its adverse effects on human health and on the environment [1]. Ambient air pollution alone kills around three million people every year, mainly from noncommunicable diseases. Only one person in ten lives in a city that complies with WHO Air quality guidelines [2].

Policies implemented at regional and national level targeting the limitation of emissions have led to acceptable air quality levels across Europe regarding some air pollutants [3], but others still raise concern, such as particulate matter, nitrogen dioxide and ozone [4].

Air pollutants are emitted by natural and anthropogenic sources; they may either be released directly (primary pollutants) or formed in the atmosphere (as secondary pollutants); they may be formed and transported over long distances or produced locally. Effective measures to decrease the impacts of air pollution require a good understanding of its sources, how pollutants are transported and transformed in the atmosphere, and how they affect humans, ecosystems, the climate, and subsequently society and the economy [5]. A regular monitoring program of air pollutants is therefore a crucial tool of successful environmental management.

Setúbal (Portugal) includes an area of high population density, anthropogenic industrial activities, traffic and protected natural areas. Moreover, the region also exhibits high levels of pollution. Several studies have already shown that local activities have a significant impact on the air quality due to: (i) emissions of air pollutants originating in industrial processes [6,7,8], (ii) dust fugitive emissions from the harbors [9,10], and (iii) intense traffic of heavy duty vehicles [11].

The present study provides a long-term assessment (ten years) of the air quality in Setúbal and aims to identify the main sources of air pollution. Evaluation of the temporal variability of several air pollutants (PM_10_, PM_2.5_, O_3_, NO, NO_2_ and NO_x_) was performed and a comprehensive characterization of particulate matter levels was conducted, along with the identification of pollution sources, using the Positive Matrix Factorization (PMF) model.

## 2. Materials and Methods

### 2.1. Study Area

This study was conducted in Setúbal (south-west Portugal), a coastal city sited where the river Sado flows into the Atlantic Ocean. Setúbal district covers an area of 230 km^2^ and it has a total population of 135,000 inhabitants [12]. The city of Setúbal is located next to two protected natural areas (Sado Estuary Reserve and Arrábida Park, which belong to the protected area Natura 2000 network) and with one of the most important industrial areas in the country. This industrial area includes: (i) different types of large industry (such as the production of fresh and dry baker’s yeast, a slaughterhouse, a powerplant, and fertilizers, pesticides, cement and chemical industries), (ii) harbors, and (iii) heavy duty vehicle traffic due to transport of raw materials and products to the harbors and industries.

### 2.2. Monitoring of Air Pollutants by Air Quality Monitoring Networks

Air quality data were obtained from three different air monitoring networks, namely from the Portuguese Environment Agency (APA-QUALAR, with 4 stations), the EDP company (two stations) and the SECIL company (three stations), for the period 2003–2012. Moreover, a field campaign was organized in this study to conduct PM sampling where two monitoring stations were established (“Industrial Mitrena” and “Quebedo”).

**Figure 1:**
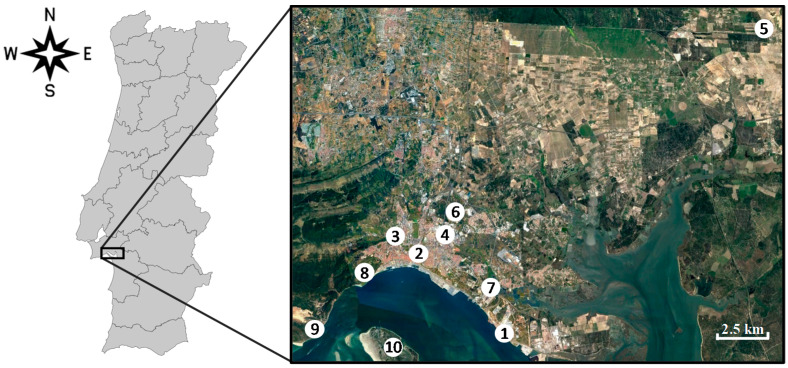
Location of the monitoring stations of the Setúbal area (Portugal): urban background (3 and 4), urban traffic (2), suburban traffic (6), rural background (5), suburban background (8 and 10) and suburban industrial (1, 7 and 9).

These monitoring stations were classified as rural background, urban background, suburban background, suburban industrial, urban traffic and suburban traffic. Figure 1 presents the location of the air quality monitoring stations and Table 1 provides a description of each monitoring station, with details of the monitored pollutants and measuring period.

These monitoring stations provided hourly data for the air pollutants NO, NO_2_, NOx, PM_10_, PM_2.5_ and O_3_. Not all monitoring stations provided data for all pollutants nor for all the study period. Different measuring/monitoring methods were applied according to the air pollutant, namely beta-attenuation for PM_10_ and PM_2.5_, chemiluminescence for NO, NO_2_ and NOx, and UV photometry for O_3_, as defined by European legislation [13].

### 2.3. Particulate Matter Sampling and Characterisation

#### 2.3.1. Sampling Sites and Methodology

PM sampling was done simultaneously in two different sampling sites within the study area and during two seasons of 2011 (winter: from 17 to 31 January–15 sampling days; summer: from 19 August to 2 September–14 sampling days). One sampling site was located at in urban traffic station (Quebedo) and the other sampling site was located in an industrial site (Mitrena). Figure 1 and Table 1 include these sampling sites, their location and other information.

Coarse and fine particulate matter were sampled using low volume Gent collectors (University of Gent, Gent, Belgium) equipped with a Stacked Filter Unit (SFU) and a PM_10_ pre impactor stage. The SFU carried two 47 mm Nuclepore® polycarbonate filters, one in each of its two different stages. Air flow rate was set to 15–16 L·min^−1^, allowing the collection of coarse particles in the first stage (particles with aerodynamic diameter (AD) between 2.5 and 10 μm-PM_2.5–10_, using a 8 µm pore size filter) and of fine particles in the second stage (particles with AD < 2.5 μm-PM_2.5_, 0.4 µm pore size filter) [14]. Filter sampling was conducted during periods of 12 h (day and night periods).

#### 2.3.2. Gravimetric Analysis

PM loads in filters were measured by gravimetry in a controlled clean room (class 10,000), with the following conditions: (20 ± 1) °C and relative humidity of (50 ± 5) %, after 48 h equilibrium. Nuclepore filters were weighted on an UMT5 Comparator balance (Mettler Toledo GmbH, 2000, Greifensee, Switzerland), an ultra-micro balance with a 0.1 µg resolution. Filter weight was measured before and after sampling and each final weight was accepted as the average of three measurements only if the variability between them was less than 5%.

#### 2.3.3. Chemical Analysis

Sampled filters were cut into two halves, with each one being used for a specific technique for a specific chemical analysis: (i) chemical elements were quantified by Instrumental Neutron Activation Analysis using the *k*_0_ methodology (*k*_0_-INAA); and (ii) water soluble ions were assessed by Ion Chromatography (IC).

Regarding *k*_0_-INAA, after being rolled up and put in a clean thin aluminum foil, each half filter was irradiated for a period of 5h in a Portuguese Research Reactor, using a thermal neutron flux of 1.03 × 10^13^ cm^−2^∙s^−1^, as established in the procedure described elsewhere [15]. After being removed from the aluminum foil, irradiated samples were stored in polyethylene containers and two gamma spectra were measured using a hyper-pure germanium detector (the first measured three days after irradiation and the second after four weeks).

For application of the *k*_0_ methodology, comparators were co-irradiated with samples, namely, 0.1% Au–Al discs. This methodology allowed the quantification of 13 chemical elements, namely As, Ce, Co, Cr, Fe, K, La, Na, Sb, Sc, Se, Sm and Zn. Blank filters were processed as regular samples and their concentrations were subtracted from the sampled filters. Quality control was done with the analysis of the reference material NIST-SRM 1633a (Coal Fly Ash) simultaneously with the samples and evaluation was performed using established procedures [16,17].

Regarding IC, a total of three anions (Cl^−^, NO_3_^−^ and SO_4_^2−^) and five cations (Na^+^, NH_4_^+^, K^+^, Mg^2+^ and Ca^2+^) were assessed using an established methodology [18]. For this, sampled and blank filters were extracted (using 5 mL of ultrapure water in an ultrasonic bath (Branson 3200, Brookfield, Connecticut, USA) for 45 min) and, afterwards, extracts were filtered using a pre-washed Whatman 41^®^ filter (Whatman International Ltd, Maidstone, England). The extract liquid filtered was then analysed by IC, using a Dionex^®^ DX500 system with an isocratic pump IP20, a conductivity detector (CD20) equipped with Peaknet^®^ software (Dionex Corporation, Sunnyvale, CA, USA). For the anionic mode, the used chromatograph had an anion guard column IonPack AG14 (Dionex Corporation, Sunnyvale, CA, USA), an analytical column IonPack AS14 and an anion suppressor ASRSR Ultra 4 mm. Using a flow rate of 1.2 mL·min^−1^, the eluent was a 3.5 mmol·dm^-3^ Na_2_CO_3_ + 1 mmol·dm^−3^ NaHCO_3_ buffer solution. For analysis of cations, the chromatograph had a CSRS 300-II-4mm cation suppressor, a guard column Ion Pack CG12 and an Ion Pack CS12 column. Using a flow rate of 1.0 mL min^−1^, the eluent was a methane-sulfonic acid (MSA) 20 mM solution. The used injection volume was 100 μL and 25 μL for anions and cations, respectively.

Measurements were conducted after the chromatograph daily calibration using calibrators with mass concentrations fit to apply the linear regression model. All data were subtracted by the blanks values.

### 2.4. Meteorological Data

Hourly meteorological data (wind direction, wind speed, precipitation, temperature and relative humidity) were measured by two automatic weather stations, one located in the monitoring station “Subestação” (operated by EDP, registering data from January 2004 to December 2009) and the other located in the monitoring station “HOSO” (operated by SECIL and registering data from January 2010 to December 2012). For the PM sampling campaigns in 2011, an automatic weather station was used to gather meteorological data during the sampling periods, located in the suburban industrial site (“Mitrena”). The wind rose and pollution dispersion maps were created using the Openair project software [19].

### 2.5. Air Quality Index

Air Quality Indexes provide for the public an easy way to understand the levels of air pollutants in their area and to gain insights regarding their associated effects on health. Ultimately, this information aims to raise the awareness of citizens towards air quality and thus to change their behavior or take mitigation measures in order to minimize their exposure. Several indexes are currently used worldwide (for example, the European Air Quality Index in Europe [20], the Air Quality Index in USA [21] or the Air Quality Index in China [22]), but no general methodology has been adopted [23]. The Portuguese Environment Agency also uses an index in order to provide information about air quality to the citizens (the QualAr Index [24]).

In order to provide an understanding of the temporal evolution of air quality in the study area, an Air Quality Index was calculated based on the 10 years’ analysis of pollutants (2003–2012). This index was defined as described in Table 2, with a total of five categories, ranging from “Very good” to “Very poor”. Three main air pollutants were considered (NO_2_, O_3_ and PM_10_), but if available two additional pollutants were also used (SO_2_ and CO). The pollutant with the worst index class, in terms of highest concentrations, was responsible for the global Air Quality Index.

### 2.6. Statistical Analysis and Source Apportionment

Statistical analysis was performed using the STATISTICA software version 13 (StatSoft Europe GmbH, Hamburg, Germany). For the analysis of the results variance, non-parametric statistics at a significance level of 0.050 were selected. Mann-Whitney tests were used to assess significant differences between datasets.

The Positive Matrix Factorization (PMF) model was used to identify the pollution sources contributing to PM levels [25]. PMF is a popular receptor model used for source apportionment studies, which decomposes the data matrix into two sub-data matrixes (factor profiles and factor contributions) without prior knowledge of the profiles of pollution sources [26]. PMF was applied to the datasets of PM sampled in “Quebedo” and “Mitrena”. Data below the limit of detection (LoD) were replaced by LoD/2 and the uncertainties were set to 5/6 of the LoD.

## 3. Results and Discussion

### 3.1. Meteorological Data

A brief summary of the meteorological data measured during the period 2004–2012 is provided in Table S1 (supplementary material). The average monthly temperature ranged from 12 °C in January/February to 27 °C in July/August. The average temperature and relative humidity (RH) in Setúbal, during the period 2004–2012, was 16.4 °C and 70.3%, respectively. The average annual accumulated precipitation was 900 mm. Rainfall was more frequent during autumn and winter.

**Figure 2:**
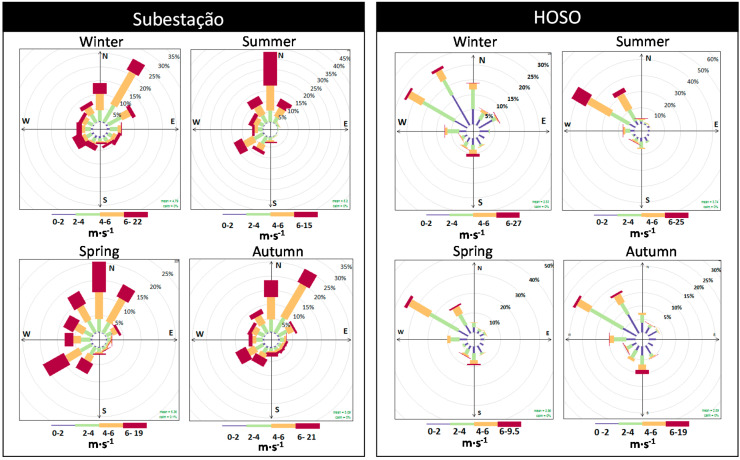
Seasonal wind roses at weather stations Subestação (**left**, during 2004–2009) and HOSO (**right**, during 2010–2012).

The wind patterns in the study area varied according to the location of the meteorological station and the season, as shown in Figure 2.

At Subestação, the main wind directions were predominantly from N and SW in summer and spring, while in winter and autumn they were from NNE. At HOSO, the predominant wind directions were from NNW and WNW. Overall, winds measured in HOSO were weaker than those registered at Subestação. This fact may be explained by HOSO’s location at the bottom of the mountain chains of Arrábida, which may protect the station from prevailing north and northwest winds.

**Figure 3:**
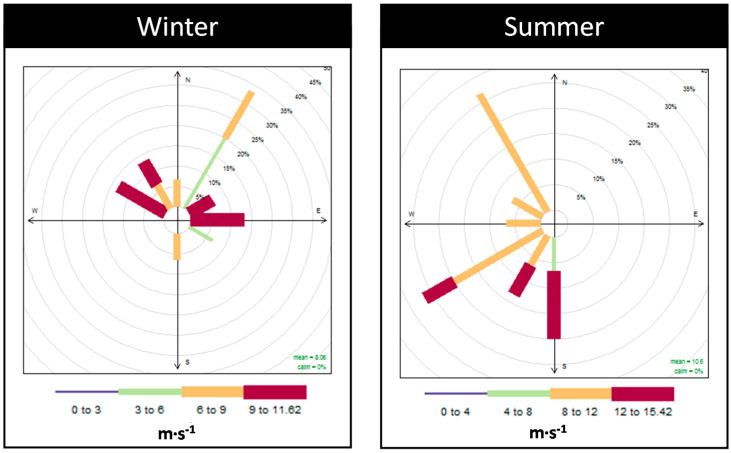
Wind roses during the PM sampling campaign in winter (**left**) and summer (**right**) seasons.

As shown in Figure 3, for the PM sampling campaigns, the prevailing winds were from NNE in winter and from NNW and WSW in summer. In winter period, a mean temperature of 12 °C and a mean relative humidity of 74% were registered, while in summer a mean temperature of 22 °C and a mean relative humidity of 61% were registered.

### 3.2. Air Quality Index (AQI)

**Figure 4:**
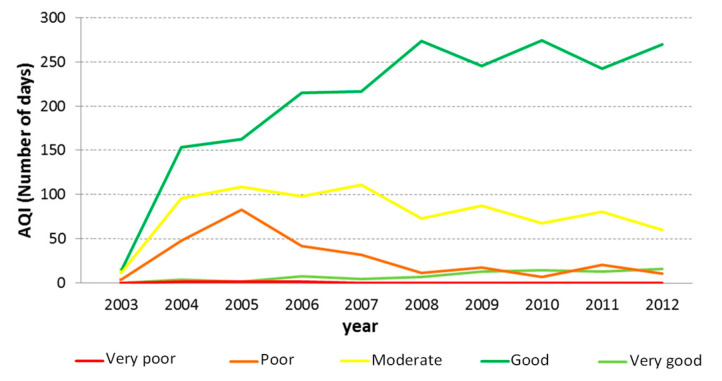
Air Quality Index registered in Setúbal area from 2003 to 2012.

Figure 4 provides the temporal evolution of the AQI in the study area, from 2003 to 2012. Overall, it is possible to observe a clear trend of better air quality indexes along the years, with the index “Good” increasing from 150 days per year in 2004 to more than 250 days per year in 2012. The index “Poor” showed a higher peak in 2005 with around 80 days per year and, afterwards, a decreasing trend with around 10 days per year in 2012. During the studied period, almost no days registered a “Very Poor” index, with the exception of a few days in the first four years. However, the number of days with a “Very good” index showed a slight increase over the years, with 2012 having around 20 days.

One possible cause for this improvement in the Air Quality Index, especially after 2007, is the reduction of energy consumption, a consequence of the world economic crisis that affected the country, a trend that has already been observed in other Portuguese cities, like Lisbon and Porto, for pollutants such as PM_10_ and NO_2_ [27]. Adding to this factor, the geographic position of the study area, which is influenced by clean air masses from the Atlantic Ocean [28], may potentiate this improvement since it contributes to good dispersion conditions of pollutants from local industrial and urban sources.

### 3.3. Temporal Patterns of Air Pollutants

#### 3.3.1. Annual Trends

**Figure 5:**
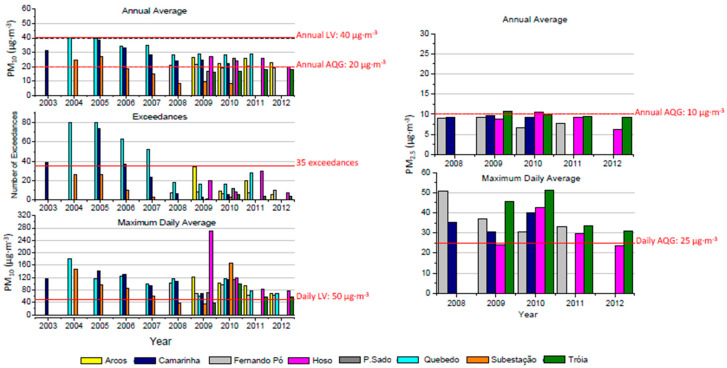
Annual average, exceedances and maximum daily average for PM_10_ (**left**) and PM_2.5_ (**right**) during the period 2003–2012 for Sétubal area. Red line stands for the established limit values (LV) or air quality guidelines (AQG).

**Figure 6:**
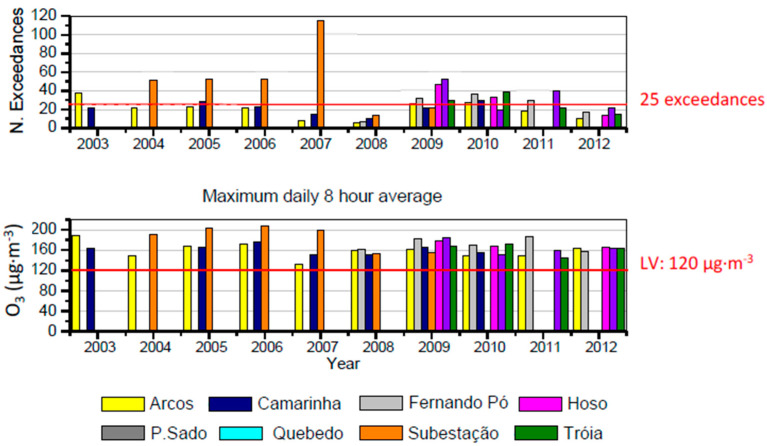
Maximum daily eight hours concentrations of ozone and number of exceedances during the period 2003–2012 at Setúbal area. Red line stands for the established limit values (LV).

**Figure 7:**
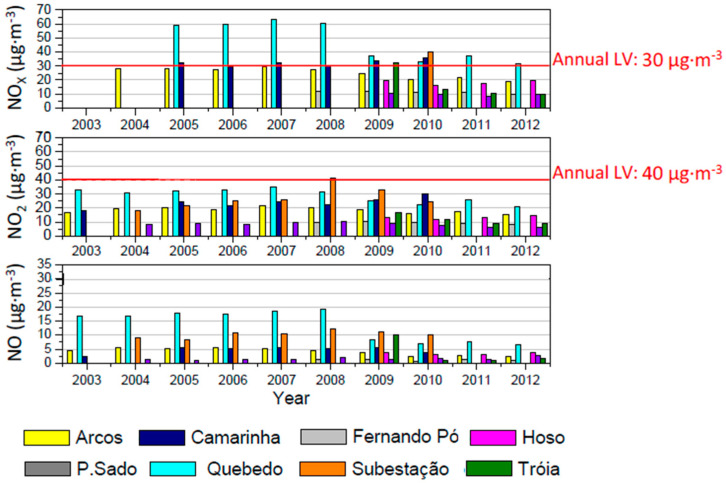
Annual concentrations of nitrogen compounds during the period 2003–2012 for Setúbal area. Red line stands for the established limit values (LV).

Figure 5, Figure 6 and Figure 7 present the annual variability of PM_2.5_ and PM_10_, O_3_ and NO, NO_2_ and NO_X_, respectively, during the studied period. When applicable, the number of exceedances taking in account the limit values and air quality guidelines established in the standards defined in Table S2 (supplementary material) are also included.

The urban traffic station located in Quebedo presented the highest annual PM_10_ concentration with a mean value of 34 µg·m^−3^ for the period 2004–2012, whereas the average PM_10_ concentration in all the studied stations was 25 µg·m^−3^. The annual averaged PM_10_ concentrations were always below the limit value of 40 μg·m^−3^, established by EU Directive 2008/50/EC. The annual threshold of 35 exceedances regarding the limit of 50 μg·m^−3^ during a 24 h period, established by EU legislation, was surpassed only in two monitoring stations: the urban background monitoring station “Camarinha” (in 2003, 2005 and 2006) and the urban traffic monitoring station “Quebedo” (from 2004 to 2007). After 2007, no monitoring stations surpassed the annual limit of 35 exceedances regarding the established daily PM_10_ concentration of 50 μg·m^−3^. However, occasionally, very high daily levels of PM_10_ were measured in several monitoring stations, for instance, the suburban industrial “HOSO” reached a daily concentration slightly above 260 μg·m^−3^ in 2009. Figure 5 shows a decreasing annual trend of the PM_10_ levels from 2005 to 2008.

Considering the annual guide value of 20 μg·m^−3^ for PM_10_ levels recommended by the World Health Organization (WHO) [29], exceedances were observed in all the studied years, for at least one of the studied monitoring stations. The suburban background monitoring station “Tróia” was the only one that presented annual values always below this guideline value (for a total of four years with data). Monitoring stations “Camarinha”, “Quebedo”, “Arcos” and “P. Sado” always presented annual values above 20 μg∙m^−3^ for all monitored years. Moreover, 71%, 60% and 75% of the monitored years above the mentioned threshold were found in the monitoring stations “Subestação”, “Fernando Pó” and “HOSO”, respectively. In 2011, a study focused on the analysis of trends of air quality in Europe from 2002 to 2011 [30] and revealed that 33% of the urban population in EU-27 lived in areas where the daily limit value for PM_10_ was exceeded and 88% of urban dwellers were exposed to PM_10_ levels that exceeded the WHO AQG for the protection of human health.

Regarding fine particulate matter (PM_2.5_), the annual limit value of 25 μg·m^−3^ defined by EU legislation was not reached in any of the studied monitoring stations. However, regarding the WHO AQG that establishes a guideline value of 10 μg·m^−3^ for the annual concentration, only two monitoring stations surpassed this value, namely “Tróia” in 2009 and “HOSO” in 2010. Regarding the maximum daily average of 25 µg·m^−3^ defined also by the WHO AQG, all monitoring stations presented higher values in all studied years (2008 to 2012), except the monitoring station “HOSO” in 2009. It is relevant to highlight the trend of a decreasing inter-annual variability of PM_10_ levels, while the levels of PM_2.5_ show a stable profile during the studied years.

Figure 6 presents the variability of ozone levels and its exceedances of the established limit value of 120 μg·m^−3^ (8 h) during the studied period. The highest concentrations were monitored in the suburban traffic monitoring station “Subestação”, with a total of 115 exceedances in 2007 surpassing the annual limit of 25. Regarding the eight hours mean, all monitoring stations presented values above the threshold of 120 μg·m^−3^ at least once per year. For the period between 2002 and 2011, in Europe 14% of the urban population was exposed to O_3_ levels above the EU target value for protecting human health [30]. Higher O_3_ concentrations are more pronounced in Mediterranean countries from southern Europe, due to the more favorable meteorological conditions for its formation such as higher biogenic emissions in summertime, higher insolation, lower deposition under hot and dry conditions and intensive recirculation of air masses [31,32].

Figure 7 presents the levels of nitrogen compounds during the studied period. The WHO AQG for NO_2_ is similar to the European annual limit value of 40 µg·m^−3^, which was surpassed only once in 2008 in the monitoring station “Subestação”. For NOx, the monitoring station “Quebedo” registered annual mean levels of almost twice the annual limit value from 2005 to 2008, and after 2008 measured levels decreased to values slightly higher than the annual limit. The monitoring stations “Camarinha” (in 2005, 2007, 2009 and 2010), “Tróia” (in 2009) and “Subestação” (in 2010) also registered values above this threshold. For NO, the annual levels at all monitoring stations were always below the value of 20 µg·m^−3^. The decrease in NOx compared to NO_2_ suggests that the proportion of NO_2_ in NOx in ambient air has increased. This can be explained by the fact that in the older diesel engines approximately 95% of NOx emissions were NO and only 5% were NO_2_. However, in the new diesel passenger cars, both engine size and exhaust after treatments (e.g., catalytic converters) increased the level of NO_2_ emissions [33].

Overall, the annual variability of the pollutants shows a decreasing trend, except for ozone. This decrease in pollutant levels is probably due to the implementation of cleaner technologies in the industry, the development of less polluting vehicles and the impact of the economic crisis that promoted the decrease of production and closure of some industrial units in the study area [27].

#### 3.3.2. Monthly Trends

**Figure 8:**
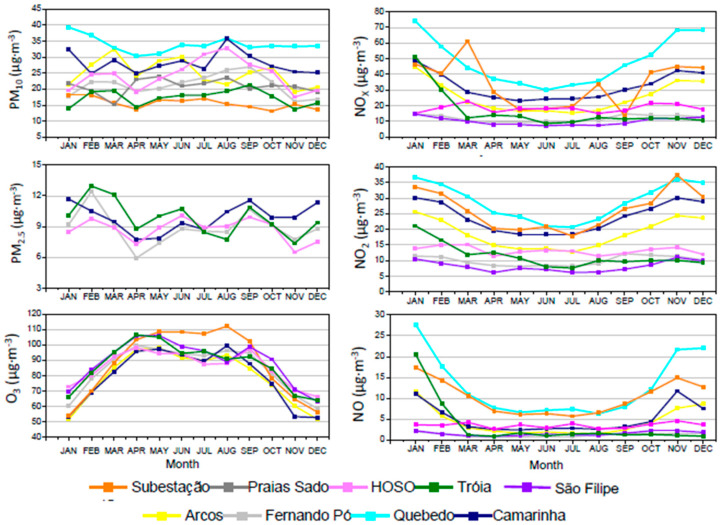
Mean monthly levels of PM_10_, PM_2.5_, O_3_, NO, NO_2_ and NO_x_ registered in the monitoring stations during the period 2003–2012 for Setúbal area.

The seasonal variability of pollutants concentrations may provide inputs regarding the processes leading to their production. Figure 8 presents the mean monthly levels of the pollutants measured in the monitoring stations between 2003 and 2012.

Ozone concentrations present a clear seasonal trend with high levels during summer. This season has ideal weather conditions for the formation of this atmospheric oxidant: warm temperatures, sunlight and high emissions of precursor pollutants (NO_x_ and volatile organic compounds - VOC) that lead to high levels of ozone [32].

The monthly variation of NO, NO_2_ and NO_x_ concentrations followed the opposite trend with lower levels during the summer. The stronger vertical atmospheric mixing in summer helps the dispersion and mixture of pollutants, which contributes to lower NO levels [34]. The apparent NO_2_ seasonal variation was probably due to NO_2_ depletion during the tropospheric O_3_ formation, which is higher in summer [35,36]. It is also important to highlight the high peak of NO_X_ levels in March registered at the monitoring station “Subestação”. For PM, the monthly average concentrations did not present a clear trend.

#### 3.3.3. Hourly Trends

**Figure 9:**
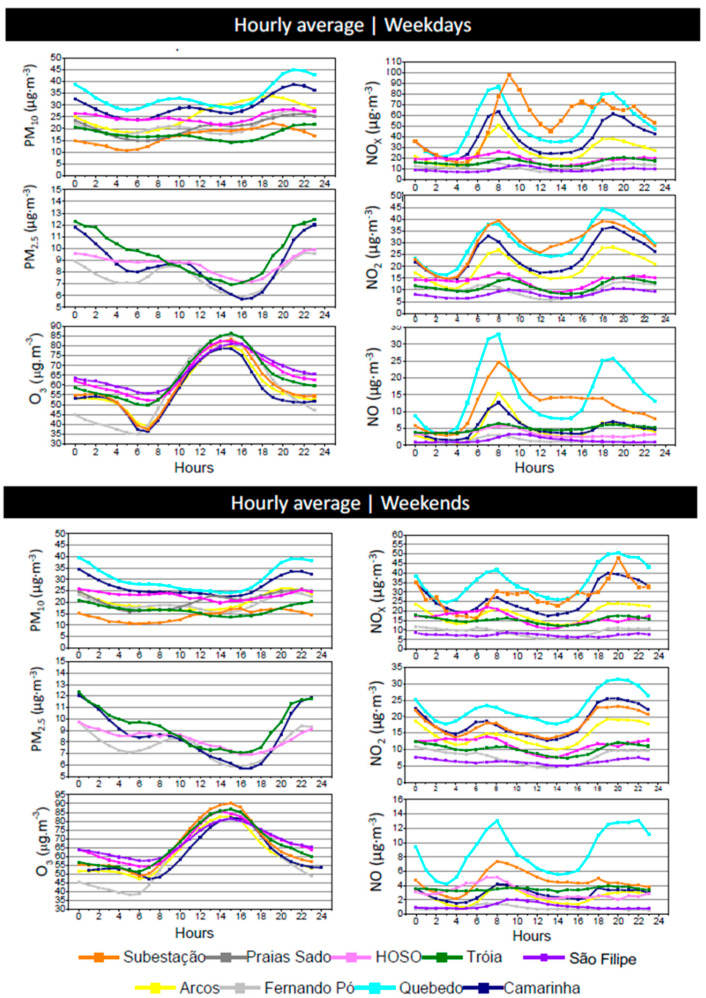
Hourly levels during weekdays (top) and weekends (bottom) of PM_10_, PM_2.5_, O_3_, NO_2_, NO_x_ and NO registered in the monitoring stations during the period 2003–2012 at Setúbal area.

The daily patterns of the studied pollutants are presented in Figure 9. Regarding particulate matter, it is possible to observe different daily profiles depending on the type of monitoring station. “Quebedo” monitoring station is defined as an urban traffic type and it showed higher concentrations of PM_10_ during vehicle peak hours, which highlights traffic influence (mainly due to resuspension [37]). However, it is also possible to observe a similar trend in the stations “Camarinha” (urban background) and “Fernando Pó” (rural background), which highlights traffic contribution to their PM levels, despite being classified as background stations.

Overall, in the monitoring stations influenced by traffic, it is possible to observe two daily peaks during weekdays for PM_10_ and PM_2.5_ concentrations: the first between 7:00 and 10:00 and the second higher one observed between 20:00 and 1:00. This behavior reflects the association of these pollutants with traffic and the poor dispersion conditions during the evening hours, which are characterized by strong atmospheric stability and light winds [38,39]. During the weekend, this pattern is not observable, indicating the lower traffic influence in this period.

The monitoring stations under the influence of industry (“HOSO” and “Praias Sado”) presented a different behavior of PM_10_ levels. PM emissions in industrial areas are a complex mixture of stationary and diffuse emissions associated with general site operations such as stocking and transportation of raw materials [40].

Traffic influence can also be confirmed regarding the daily pattern during weekdays of NO, NO_2_ and NOx where a high level related to traffic peak hours could be found. This is mainly visible in vehicle peak hours at monitoring stations with traffic influence, such as “Quebedo” (urban traffic) and “Subestação” (suburban traffic), and also at monitoring stations considered as urban background (“Camarinha” and “Arcos”), and this is clearly related to engine combustion emissions [41].

NO concentrations were higher in “Quebedo”, “Subestação”, “Arcos” and “Camarinha” than in the other stations. “Quebedo” and “Arcos” presented a strong correlation with each other (0.82). These stations have two daily peaks: the first between 7:00 and 9:00 and the second between 17:00 and 19:00 reflecting the morning and evening rush hours. The trend in “Subestação” was characterized by only one NO peak between 7:00 and 9:00 and two NO_2_ peaks in the morning and afternoon, which may indicate a larger influence of the traffic source and NO emissions during the morning that resulted in lower NO_2_/NOx ratios. In the afternoon, the site was less affected by traffic, which increased the NO_2_/NOx ratio due to the oxidation of NO.

A relevant difference between weekdays and weekend is possible to observe for the levels of NO, NO_2_ and NO_x_. During weekends the levels go down by half during the peak hours in the urban stations, when compared with weekdays, which highlights the traffic source of these pollutants.

Regarding ozone, the hourly trends indicate that this pollutant is directly related to the presence of solar radiation, showing lower values in the evening. Since O_3_ is a secondary pollutant, which means that it is not emitted directly into the atmosphere, its production is achieved in the presence of sunlight by photochemical reactions between NO_x_ and VOCs, explaining the observed pattern. Overall, O_3_ levels did not differ significantly among the studied monitoring stations.

### 3.4. Characterisation of Particulate Matter

In order to understand the pollution sources of particulate matter affecting the study area, a sampling campaign of fine (PM_2.5_) and coarse (PM_2.5–10_) particles was conducted during two different seasons (winter and summer) in 2011 at two different study sites. This section presents the seasonal evaluation of PM levels, their chemical characterization and the source apportionment.

#### 3.4.1. Mass Concentrations

**Figure 10:**
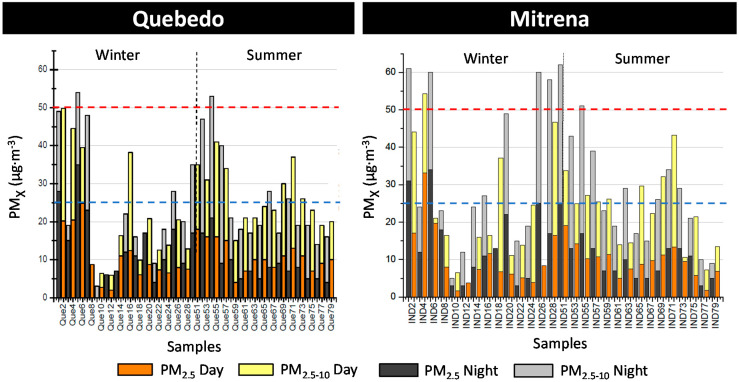
PM concentrations (fine and coarse fractions) sampled in “Quebedo” (**left**) and “Mitrena” (**right**) monitoring stations, during winter and summer. Red and blue dash lines stand for the daily limit value of PM_10_ (50 µg·m^−3^) and annual limit value of PM_2.5_ (25 µg·m^−3^), respectively, established by European legislation.

PM levels at both studied monitoring stations (“Quebedo” and “Mitrena”) during daytime and nighttime are shown in Figure 10 for winter and summer, along with their compliance with European legislation.

In the urban monitoring station “Quebedo”, levels of fine particles ranged from 2 to 35 µg·m^−3^ with a mean value of (12.7 ± 8.1) µg·m^−3^ during the winter period, while during the summer period PM_2.5_ levels ranged from 4 to 21 µg·m^−3^ with a mean value of (9.9 ± 4.5) µg·m^−3^. Only two exceedances of the limit value of 25 µg·m^−3^ were registered in winter and both occurred during the night period. Regarding the coarse fraction, mean levels of (11.7 ± 8.2) µg·m^−3^ and (16.2 ± 6.7) µg·m^−3^ were registered during winter and summer, respectively. PM_10_ levels ranged from 3 to 55 µg·m^−3^ during winter with a mean level of 22 µg·m^−3^ and from 14 to 53 µg·m^−3^ during summer with a mean level of 26 µg·m^−3^. Only two exceedances to the daily PM_10_ limit value of 50 µg·m^−3^ were registered, one in each sampling season, but both during the night period.

In the industrial monitoring station “Mitrena”, PM_2.5_ levels ranged from 2 to 36 µg·m^−3^ with a mean value of (13.0 ± 9.6) µg·m^−3^ during winter, while during summer levels ranged from 2 to 19 µg·m^−3^ with a mean value of (9.4 ± 4.1) µg·m^−3^. These levels were very similar to the ones registered in the “Quebedo” monitoring station. Regarding the coarse fraction, the mean levels registered in Mitrena (16.4 ± 11.7) µg·m^−3^ during winter were higher than in Quebedo, principally during the night. This can be explained by the fact that in the industrial site the PM emissions from industry (mainly fugitive emissions) occur during 24 h, while the influence of non-exhaust traffic emissions in the urban area occurs mainly during the day. PM_10_ levels ranged from 4 to 62 µg·m^−3^ during winter with a mean value of 29 µg·m^−3^, while in summer PM_10_ levels ranged from 7 to 52 µg·m^−3^ with a mean value of 25 µg·m^−3^. In winter, six exceedances of the daily PM_10_ limit value of 50 µg·m^−3^ were registered (five during the night period and one during daytime), while during summer only one exceedance was recorded (during the night period).

#### 3.4.2. Chemical Characterisation

Table 3 and Table 4 present the characterization of both fractions of particulate matter (PM_2.5_ and PM_2.5-10_) sampled in “Quebedo” and “Mitrena” monitoring stations, respectively, regarding the mass concentrations and the content of chemical elements and water soluble ions.

For both monitoring stations, the most abundant ions in the fine fraction (PM_2.5_) were SO_4_^2−^, NO_3_^−^ and NH_4_^+^, which are associated with secondary aerosols [42], resulting from emissions of industry activities and traffic [43]. In the coarse fraction (PM_2.5–10_), the main components were Cl^−^ and Na^+^, which are typically associated with a sea salt source [26], and Ca^2+^, which is associated with a crustal origin [42].

The Mann-Whitney test showed that in “Quebedo” only Ca^2+^, in both fractions, and NH_4_^+^, in the coarse fraction, presented significant differences between day and night. The higher concentrations of Ca^2+^ during the day are probably due to dust re-suspension associated with traffic [37]. NH_4_^+^ presented higher concentrations overnight. In the industrial monitoring station “Mitrena”, no significant differences between day and night concentrations were found.

During summer in “Quebedo”, considerably high concentrations were registered for: (1) SO_4_^2−^, due to the strong solar radiation that increases temperature and stimulates the formation of OH radicals, thus promoting the formation of secondary sulphates [44]; (2) La and Sm in the coarse fraction, which are associated with increased dust re-suspension in the dry period; and (3) NO_3_^−^ in the coarse fraction, which can be partly attributed to the reaction of HNO_3_ with mineral species, such as calcium carbonate and sea salt to form Ca(NO_3_)_2_ and NaNO_3_, respectively. These reactions are prevalent in the warm season, while in winter NO_3_^−^ preferentially reacts with NH_3_ to form NH_4_NO_3_ [45]. During winter, high concentrations of NO_3_^−^ were observed in the fine fraction and a strong contribution from the wood burning used in dwellings for house heating was also observed corroborated by high concentrations of K and Sb observed in both fractions and as in the fine fraction when compared with summer [26,46].

In the “Mitrena” monitoring station, an increase of NO_3_^−^ levels in the fine fraction during winter and in the coarse faction during the summer was observed. SO_4_^2−^ only presented high concentrations in summer for the coarse fraction. As, Sb, Zn and K showed significantly high concentrations in winter for the fine fraction.

Figure S1 (supplementary material) provides the comparison between the PM levels and its components found in both monitoring stations, which allows us to understand the existence of local or non-local sources for PM. High correlations between both monitoring stations, along with similar levels in both, were found regarding SO_4_^2−^, NO_3_^−^ and NH_4_^+^, which are from secondary aerosols, along with the ions Na^+^ and Cl^−^, which are associated with sea salt spray [42]. These correlations suggest that these species and elements are from non-local sources. Low correlations were found for Zn, Sb, As, Cl^–^, Ca^2+^, Fe, Sm, K^+^ and Cr between both monitoring stations, revealing that there were local sources contributing to the air concentrations of these species. Cr was not associated with a preferential sampling station, indicating the existence of multiple sources for this element. As, Zn, Cl^−^, Ca^2+^ and K^+^ presented higher concentrations in “Mitrena”, indicating the existence of local sources for these species. Sb, Fe and Sm had high levels in “Quebedo” urban traffic monitoring station, probably due to the contribution of vehicles traffic, namely due to tire and break wear and road dust re-suspension [42].

#### 3.4.3. Identification of Emission Sources

**Figure 11:**
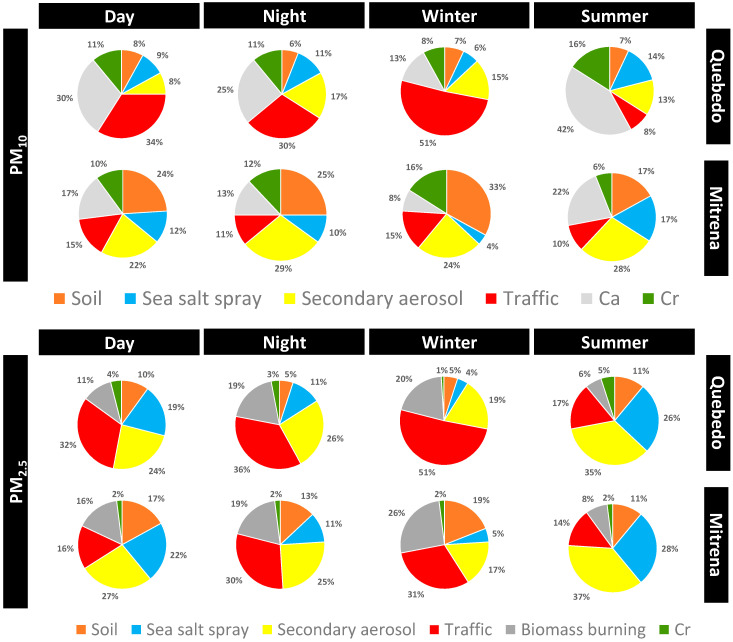
Source contributions (in %) to PM_10_ (**top**) and PM_2.5_ (**bottom**) mass sampled in the monitoring stations “Quebedo” and “Mitrena”.

In order to identify and assess the contribution of emission sources to the sampled PM levels, a source apportionment study was conducted using the PMF model. Figure 11 presents the contribution of the different sources for the PM levels, where six main chemical sources were identified in both PM_2.5_ and PM_10_. Figure S2 (supplementary material) provides the mass contribution of the assessed sources to the PM levels of each sampling period.

Regarding PM_2.5_, the six main chemical profiles/factors assessed were the following:

(1) Factor 1 was associated with soil since a high association with typical soil elements was found, namely Ca, Sc, Sm and La [42]. This crustal source contributed, on average, to 8% of the total PM_2.5_ mass in “Quebedo” and 15% in “Mitrena”. The contribution of this source was higher during daytime probably due to the resuspension related to traffic and due to an increase of the activities dealing with materials handling in the industrial area (“Mitrena”).
(2) Factor 2 presented high associations between the water-soluble ions NH_4_^+^ and SO_4_^2−^ and the elements Co and Se, which are associated with secondary aerosols [47]. This source made an average contribution of 26% to the total PM_2.5_ mass in both monitoring stations. In both sites, a higher contribution was found in summer than in winter and contribution during day and night was similar.
(3) Factor 3 was associated with sea salt spray since a high association was found between the water soluble ions Na^+^, Mg^2+^ and Cl^−^, which are associated with this source [42]. This source contributed around 15% to the total PM_2.5_ mass in both monitoring stations, with a higher contribution during summer probably due to the more intense sea breeze and the dominant S/SW winds registered during that season (Figure 3).
(4) Factor 4 was characterized by water soluble ions K^+^ and Cl^-^ and by As, which are associated with wood burning [48]. This source made a high contribution during the winter in both monitoring stations due to the use of wood burning for house heating. Overall, this source contributed, on average, 15% and 17% to the total PM_2.5_ in “Quebedo” and “Mitrena”, respectively.
(5) Factor 5 was associated with a traffic source due to the high association between Sb, NO_3_^-^, As and Fe. NO_3_^-^ is associated with car emissions and As and Sb are typically from mechanical abrasion of brakes and tires [37,42,49]. Fe may be associated with dust resuspension since this element is typically associated with crustal sources [42]. The traffic contribution was higher in “Quebedo” (34%) than in “Mitrena” monitoring station. In both monitoring stations, the contribution of this traffic source was higher in winter than in summer.
(6) Factor 6 was characterized by Cr, which represents the industrial contribution [50] that was, on average, 3% and 2% for the monitoring stations “Quebedo” and “Mitrena”, respectively.

For PM_10_, the same sources were identified, with the exception of the contribution of wood burning and with the identification of a new source of Ca^2+^. This calcium source is probably from the cement industry [41] that exists in the area of Setúbal. Overall, the contribution of this source to the total PM_10_ was 27% and 15% in monitoring stations “Quebedo” and “Mitrena”, respectively. The traffic contributed to 32% of PM_10_ load in “Quebedo” and 13% in “Mitrena”. The industrial emissions characterized by Cr and sea salt spray contributed, on average, to around 11% to the total PM_10_ in both sites. The secondary aerosols contributed to, on average, 13% and 26% of the total PM_10_ in “Quebedo” and “Mitrena”, respectively.

## 4. Conclusions

This study allowed an understanding of the temporal evolution of the air quality during a period of ten years (2003–2012) in Setúbal, an urban area with a high influence of industrial activities. Overall, the air quality index has been improving during the studied period. Setúbal has a set of climate variables, which favors good dispersion of pollutants and, ultimately, confer good air quality in this region despite strong industrial emissions.

With the exception of ozone, all pollutants have demonstrated a decreasing trend, probably due to the implementation of cleaner technologies in the industries, the development of less polluting vehicles; during the study period, the global economic crisis situation also had an impact on the region, which promoted a decrease in production and the closure of some industrial units. However, despite this trend, some pollutants still presented exceedances of the European and WHO guidelines, namely, particulate matter (PM_2.5_ and PM_10_), NOx and ozone.

Characterization of PM_2.5_ and PM_10_ levels in the area allowed the identification of the main sources contributing to local PM levels, namely, traffic, industry and wood burning, which should be addressed by specific mitigation measures in order to minimize their impact on local air quality.

## Figures and Tables

**Table 1:** Description of the monitoring stations located in Setúbal region (regular monitoring stations and field study stations).

Network	#ID	Stations	Coordinates		Altitude (m)	Type	Pollutants Monitored	Sampling Period for Each Pollutant
			Latitude	Longitude				
This study	1	INDUSTRIAL MITRENA	38°29’48’’N	08°49’58’’W	–	Suburban Industrial	PM_2.5–10_ and PM_2.5_ mass and chemical composition	PM: 2011
								chemical constituents: 2011
	2	QUEBEDO	38°31’27’’N	08°53’39’’W	16	Urban Traffic	PM_2.5–10_ and PM_2.5_ mass and chemical composition	PM: 2011
								chemical constituents: 2011
APA–QUALAR		QUEBEDO	38°31’27’’N	08°53’39’’W	16	Urban Traffic	NO, NO_2_, NO_X_, PM_10_	NO, NO_2_: 2003–2012
	2							NO_x_: 2005–2012
								PM_10_: 2004–2011
	3	ARCOS	38°31’46’’N	08°53’39’’W	2	Urban Background	NO, NO_2_, NO_X_, O_3_, PM_10_	NO, NO_2_, O_3_: 2003–2012
								NOx: 2004–2012
								PM_10_: 2009–2012
	4	CAMARINHA	38°31’50’’N	08°52’23’’W	15	Urban Background	NO, NO_2_, NO_X_, PM_2.5_, PM_10_, O_3_	NO, NO_2_, O_3_, PM_10_: 2003
								NOx: 2005–2010
								PM_2.5_: 2008–2010
	5	FERNANDO PÓ	38°38’08’’N	08°41’26’’W	57	Rural Background	NO, NO_2_, NO_X_, O_3_, PM_2.5_, PM_10_	2008–2011
EDP	6	SUBESTAÇÃO	38°32’08’’N	08°51’44’’W	30	Suburban Traffic	NO, NO_2_, NO_X_, PM_10_, O_3_	NO, NO_2_, O_3_, PM_10_: 2004–2010
								NOx: 2010
	7	PRAIAS SADO	38°31’05’’N	08°50’15’’W	–	Suburban Industrial	PM_10_	PM_10_: 2009–2010
SECIL	8	SÃO FILIPE	38°30’55’’N	08°54’40’’W	110	Suburban Background	NO, NO_2_, NO_X_, O_3_	NO, NO_2_: 2004–2012
								NOx, O_3_: 2009–2012
	9	HOSO	38°29’31’’N	08°56’02’’W	–	Suburban Industrial	NO, NO_2_, NO_X_, PM_2.5_, PM_10_, O_3_	NO, NO_2_, NO_X_, O_3_, PM_2.5_: 2009–2012
								PM_10_: 2010–2012
	10	TRÓIA	38°28’45’’N	08°53’20’’W	3	Suburban Background	NO, NO_2_, NO_X_, PM_2.5_, PM_10_, O_3_	2009–2012

**Table 2:** Air Quality Index categories and their pollutants range (values in µg∙m^−3^).

Index Class	Mandatory Pollutant			Auxiliary Pollutant	
	NO_2_ (1 h)	O_3_ (1 h)	PM_10_ (24 h)	SO_2_ (1 h)	CO (8 h)
Very good	[0–99]	[0–59]	[0–19]	[0–139]	[0–4999]
Good	[100–139]	[60–119]	[20–34]	[140–209]	[5000–6999]
Moderate	[140–199]	[120–179]	[35–49]	[210–349]	[7000–8499]
Poor	[200–399]	[180–239]	[50–119]	[350–499]	[8500–9999]
Very poor	≥400	≥240	≥120	≥500	≥10,000

**Table 3:** Characterization of PM_2.5_ and PM_2.5-10_ sampled at “Quebedo” urban traffic station, regarding mass concentration and contents of chemical elements and water soluble ions. Ion balance stands for the ratio of cations to anions and LoD stands for Limit of Detection.

Parameter	Unit	Annually		Winter						Summer					
		PM_2.5_	PM_2.5–10_	PM_2.5_			PM_2.5–10_			PM_2.5_			PM_2.5–10_		
				24 h	Day	Night	24 h	Day	Night	24 h	Day	Night	24 h	Day	Night
PM_X_	µg·m^−3^	11.3 ± 6.6	14.1 ± 7.7	12.7 ± 8.1	10.4 ± 6.8	15.0 ± 8.9	11.7 ± 8.2	11.8 ± 9.2	11.7 ± 7.4	9.87 ± 4.47	11.0 ± 3.9	8.68 ± 4.85	16.2 ± 6.7	15.6 ± 4.6	16.8 ± 8.5
Cl^−^	ng·m^−3^	195 ± 181	971 ± 974	195 ± 200	154 ± 142	234 ± 241	444 ± 504	472 ± 565	416 ± 453	195 ± 162	220 ± 176	168 ± 149	1480 ± 1050	1100 ± 700	1890 ± 1230
NO_3_^−^		1070 ± 1130	1070 ± 790	1620 ± 1330	1160 ± 910	2060 ± 1540	858 ± 715	869 ± 834	847 ± 614	487 ± 333	582 ± 393	392 ± 237	1290 ± 820	1240 ± 840	1330 ± 820
SO_4_^2−^		1230 ± 990	392 ± 326	907 ± 699	847 ± 665	964 ± 748	320 ± 312	317 ± 310	322 ± 325	1550 ± 1140	1490 ± 800	1620 ± 1430	465 ± 328	372 ± 234	564 ± 390
Na^+^		239 ± 241	831 ± 728	74.6 ± 59.8	75.2 ± 60.9	74.1 ± 61.4	340 ± 337	340 ± 350	341 ± 336	387 ± 247	419 ± 263	353 ± 233	1310 ± 690	1060 ± 580	1570 ± 720
NH_4_^+^		519 ± 472	53.3 ± 45.9	619 ± 558	474 ± 446	753 ± 631	61.6 ± 47.0	50.9 ± 41.1	72.2 ± 51.6	419 ± 346	419 ± 314	418 ± 390	45.0 ± 44.0	28.8 ± 22.4	61.2 ± 54.4
K^+^		126 ± 141	74 ± 102	187 ± 172	147 ± 107	224 ± 213	66.0 ± 60.6	68.0 ± 60.9	64.2 ± 62.3	64.4 ± 57.5	57.3 ± 31.0	71.9 ± 77.3	81 ± 132	48.6 ± 26.6	116 ± 186
Mg^2+^		23.5 ± 21.2	85.9 ± 64.6	9.94 ± 5.90	10.2 ± 5.58	9.72 ± 6.37	43.8 ± 29.6	46.2 ± 31.6	41.4 ± 28.4	37.0 ± 22.4	41.1 ± 22.8	32.5 ± 21.9	128 ± 63	111 ± 57	146 ± 66
Ca^2+^		210 ± 185	836 ± 829	192 ± 204	227 ± 228	150 ± 173	957 ± 1060	1210 ± 1250	708 ± 795	225 ± 169	266 ± 175	177 ± 156	724 ± 511	786 ± 329	658 ± 660
Ion balance	n/a	1.65 ± 2.22	2.25 ± 1.22	1.21 ± 0.23	1.31 ± 0.18	1.11 ± 0.24	2.71 ± 1.40	3.23 ± 1.73	2.35 ± 0.93	2.08 ± 3.10	2.71 ± 4.27	1.40 ± 0.27	1.80 ± 0.79	1.88 ± 0.47	1.71 ± 1.00
As	ng·m^−3^	0.46 ± 0.44	0.17 ± 0.13	0.63 ± 0.41	0.67 ± 0.45	0.58 ± 0.39	0.16 ± 0.12	0.18 ± 0.14	0.14 ± 0.10	0.04 ± 0.04	0.04 ± 0.04	0.04 ± 0.06	0.22 ± 0.18	0.17 ± 0.22	0.32
Ce		0.35 ± 0.24	0.28 ± 0.14	0.35 ± 0.25	0.40 ± 0.29	0.30 ± 0.21	0.25 ± 0.13	0.21 ± 0.12	0.32 ± 0.16	0.35 ± 0.26	0.38 ± 0.36	0.31 ± 0.08	n.a.	n.a.	<LoD
Co		0.09 ± 0.12	5.19 ± 13.3	0.13 ± 0.17	0.13 ± 0.20	0.11 ± 0.11	0.05 ± 0.05	0.05 ± 0.06	0.05 ± 0.05	0.07 ± 0.05	0.07 ± 0.06	0.07 ± 0.05	18.6 ± 21.1	6.18 ± 8.12	37.1 ± 22.2
Cr		3.55 ± 2.72	7.93 ± 5.13	1.29 ± 0.99	1.10 ± 0.78	1.61 ± 1.41	5.08 ± 3.16	4.94 ± 2.80	5.22 ± 3.58	4.41 ± 2.68	4.86 ± 2.48	3.99 ± 2.90	13.2 ± 3.62	12.7 ± 2.50	13.6 ± 4.32
Cs		0.68 ± 2.61	0.06 ± 0.01	0.06 ± 0.04	0.07 ± 0.03	0.05 ± 0.06	0.06 ± 0.01	0.06 ± 0.01	<LoD	1.09 ± 3.36	0.07 ± 0.09	2.12 ± 4.72	<LoD	<LoD	<LoD
Fe		138 ± 96	286 ± 236	153 ± 96	159 ± 88	147 ± 105	294 ± 262	348 ± 278	232 ± 238	121 ± 95	138 ± 113	99.9 ± 66.9	254 ± 95	293 ± 102	201 ± 66
K		137 ± 122	99.0 ± 62.8	184 ± 130	154 ± 117	212 ± 138	89.2 ± 66.5	100 ± 73	77.9 ± 59.7	61.7 ± 49.2	62.3 ± 46.5	60.9 ± 55.6	119 ± 51	106 ± 37	133 ± 65
La		0.06 ± 0.06	0.15 ± 0.13	0.06 ± 0.03	0.06 ± 0.04	0.05 ± 0.02	0.09 ± 0.09	0.10 ± 0.10	0.09 ± 0.09	0.07 ± 0.08	0.06 ± 0.09	0.07 ± 0.07	0.23 ± 0.14	0.23 ± 0.17	0.23 ± 0.12
Na		194 ± 181	697 ± 618	65.7 ± 47.0	61.1 ± 39.0	69.9 ± 54.5	302 ± 304	301 ± 294	303 ± 324	322 ± 175	348 ± 181	293 ± 170	1120 ± 590	852 ± 516	1410 ± 540
Sb		0.64 ± 0.46	0.71 ± 0.71	0.82 ± 0.51	0.82 ± 0.39	0.83 ± 9.61	0.95 ± 0.76	1.18 ± 0.76	0.73 ± 0.72	0.43 ± 0.26	0.41 ± 0.21	0.44 ± 0.32	0.23 ± 0.14	0.26 ± 0.17	0.18 ± 0.08
Sc		0.02 ± 0.02	0.05 ± 0.04	0.01 ± 0.01	0.01 ± 0.01	0.01 ± 0.01	0.02 ± 0.02	0.03 ± 0.02	0.02 ± 0.01	0.02 ± 0.03	0.02 ± 0.03	0.02 ± 0.02	0.06 ± 0.04	0.07 ± 0.03	0.06 ± 0.04
Se		0.29 ± 0.23	0.07 ± 0.05	0.30 ± 0.27	0.20 ± 0.21	0.37 ± 0.30	0.07 ± 0.05	0.02 ± 0.02	0.10 ± 0.04	0.29 ± 0.20	0.24 ± 0.17	0.34 ± 0.22	<LoD	<LoD	<LoD
Sm		0.01 ± 0.01	0.04 ± 0.06	0.01 ± 0.00	0.01 ± 0.01	0.01 ± 0.00	0.02 ± 0.02	0.02 ± 0.02	0.02 ± 0.01	0.02 ± 0.02	0.02 ± 0.03	0.01 ± 0.01	0.07 ± 0.10	0.03 ± 0.01	0.10 ± 0.13
Zn		11.0 ± 12.0	13.9 ± 14.3	13.4 ± 15.8	15.2 ± 14.0	11.8 ± 17.4	13.2 ± 14.1	12.7 ± 12.0	13.8 ± 17.3	8.44 ± 4.77	8.94 ± 5.38	7.93 ± 4.22	19.8 ± 17.9	19.8 ± 17.9	<LoD

**Table 4:** Characterization of PM_2.5_ and PM_2.5–10_ sampled at “Mitrena” suburban industrial station, regarding mass concentration and contents of chemical elements and water soluble ions. Ion balance stands for the ratio of cations to anions and LoD stands for Limit of Detection.

Parameter	Unit	Annually		Winter						Summer					
		PM_2.5_	PM_2.5–10_	PM_2.5_			PM_2.5-10_			PM_2.5_			PM_2.5–10_		
				24 h	Day	Night	24 h	Day	Night	24 h	Day	Night	24 h	Day	Night
PM_X_	µg·m^−3^	11.2 ± 7.5	15.7 ± 10.0	13.0 ± 9.6	10.6 ± 8.2	15.3 ± 10.3	16.4 ± 11.7	13.8 ± 9.7	18.8 ± 12.9	9.44 ± 4.10	9.69 ± 4.03	9.18 ± 4.16	15.1 ± 8.1	13.8 ± 9.7	16.9 ± 8.6
Cl^−^	ng·m^−3^	456 ± 685	1050 ± 900	437 ± 692	265 ± 243	597 ± 917	479 ± 552	402 ± 471	550 ± 613	476 ± 689	308 ± 218	656 ± 949	1620 ± 820	1320 ± 740	1940 ± 760
NO_3_^−^		917 ± 920	996 ± 735	1320 ± 1120	907 ± 752	1700 ± 1280	670 ± 495	574 ± 471	760 ± 500	519 ± 369	556 ± 415	480 ± 303	1320 ± 800	1220 ± 740	1430 ± 840
SO_4_^2−^		1570 ± 1120	726 ± 473	1310 ± 1010	1310 ± 1110	1310 ± 910	613 ± 494	556 ± 392	666 ± 573	1820 ± 1170	1790 ± 1100	1860 ± 1250	840 ± 430	751 ± 469	936 ± 355
Na^+^		294 ± 371	751 ± 662	88.2 ± 66.4	98.4 ± 72.0	78.8 ± 58.6	314 ± 343	281 ± 298	344 ± 379	499 ± 434	504 ± 267	494 ± 569	1190 ± 610	1040 ± 610	1350 ± 570
NH_4_^+^		534 ± 483	136 ± 224	552 ± 568	452 ± 524	646 ± 593	124 ± 152	83.0 ± 64.2	161 ± 198	516 ± 388	451 ± 341	585 ± 425	149 ± 280	98 ± 139	203 ± 374
K^+^		146 ± 151	146 ± 117	201 ± 196	170 ± 101	230 ± 254	138 ± 83	123 ± 65	152 ± 95	90.8 ± 40.7	101 ± 46	80.0 ± 29.2	155 ± 144	147 ± 90	163 ± 188
Mg^2+^		35.0 ± 63.5	93 ± 103	29.5 ± 88.1	16.8 ± 9.8	41 ± 123	70 ± 130	48.4 ± 39.7	90 ± 176	39.9 ± 19.5	44.0 ± 21.3	35.5 ± 15.8	117 ± 61	98.0 ± 56.5	137 ± 60
Ca^2+^		280 ± 261	1080 ± 1180	331 ± 313	334 ± 294	329 ± 332	1470 ± 1480	1150 ± 920	1780 ± 1820	229 ± 187	288 ± 208	166 ± 130	684 ± 565	694 ± 559	674 ± 572
Ion balance	n/a	1.29 ± 0.57	2.33 ± 1.61	1.28 ± 0.75	1.40 ± 0.64	1.19 ± 0.84	3.35 ± 1.74	3.52 ± 1.80	3.20 ± 1.73	1.29 ± 0.32	1.38 ± 0.36	1.20 ± 0.26	1.35 ± 0.49	1.41 ± 0.62	1.29 ± 0.31
As	ng·m^−3^	0.87 ± 3.14	0.66 ± 1.64	1.49 ± 4.38	2.17 ± 6.06	0.85 ± 0.85	0.30 ± 0.37	0.33 ± 0.45	0.28 ± 0.26	0.25 ± 0.26	0.26 ± 0.27	0.24 ± 0.24	1.01 ± 2.25	0.57 ± 1.01	1.49 ± 3.04
Ce		0.11 ± 0.12	0.33 ± 0.31	0.13 ± 0.13	0.12 ± 0.10	0.14 ± 0.16	0.39 ± 0.39	0.30 ± 0.26	0.47 ± 0.47	0.09 ± 0.11	0.10 ± 0.12	0.08 ± 0.10	0.28 ± 0.22	0.24 ± 0.18	0.32 ± 0.24
Co		0.04 ± 0.05	0.09 ± 0.07	0.04 ± 0.05	0.04 ± 0.07	0.03 ± 0.03	0.09 ± 0.08	0.10 ± 0.09	0.08 ± 0.06	0.04 ± 0.04	0.04 ± 0.03	0.04 ± 0.04	0.08 ± 0.06	0.07 ± 0.06	0.10 ± 0.06
Cr		2.31 ± 1.60	4.27 ± 5.27	2.56 ± 1.49	2.74 ± 1.66	2.40 ± 1.28	6.62 ± 6.25	4.46 ± 3.32	8.64 ± 7.63	2.06 ± 1.70	2.20 ± 1.68	1.90 ± 1.70	1.92 ± 2.45	2.08 ± 2.41	1.75 ± 2.49
Fe		91 ± 101	296 ± 484	61.3 ± 38.2	64.9 ± 37.0	58.0 ± 38.9	177 ± 114	185 ± 106	170 ± 120	121 ± 132	111 ± 121	132 ± 144	416 ± 660	253 ± 248	589 ± 897
K		123 ± 119	146 ± 92	187 ± 137	168 ± 130	205 ± 142	146 ± 97	154 ± 93	139 ± 100	59.4 ± 36.7	62.6 ± 28.1	56.0 ± 44.3	146 ± 89	144 ± 84	147 ± 94
La		0.11 ± 0.10	0.43 ± 0.56	0.14 ± 0.12	0.14 ± 0.12	0.13 ± 0.12	0.63 ± 0.71	0.41 ± 0.41	0.83 ± 0.87	0.08 ± 0.06	0.06 ± 0.04	0.09 ± 0.07	0.23 ± 0.21	0.18 ± 0.16	0.29 ± 0.24
Na		271 ± 293	786 ± 642	98.7 ± 68.8	105 ± 73	93.0 ± 63.2	381 ± 389	354 ± 354	406 ± 419	443 ± 329	434 ± 257	452 ± 396	1190 ± 590	1050 ± 570	1340 ± 570
Sb		0.47 ± 0.42	0.27 ± 0.20	0.67 ± 0.50	0.49 ± 0.25	0.84 ± 0.62	0.31 ± 0.23	0.28 ± 0.20	0.33 ± 0.25	0.28 ± 0.18	0.26 ± 0.13	0.30 ± 0.23	0.22 ± 0.15	0.22 ± 0.11	0.23 ± 0.18
Sc		0.01 ± 0.01	0.06 ± 0.07	0.01 ± 0.01	0.01 ± 0.01	0.01 ± 0.01	0.08 ± 0.09	0.05 ± 0.05	0.10 ± 0.10	0.01 ± 0.01	0.01 ± 0.01	0.01 ± 0.01	0.04 ± 0.03	0.04 ± 0.03	0.04 ± 0.04
Se		0.37 ± 0.23	0.14 ± 0.13	0.33 ± 0.16	0.29 ± 0.15	0.37 ± 0.16	0.06 ± 0.08	0.05 ± 0.08	0.07 ± 0.07	0.40 ± 0.28	0.38 ± 0.28	0.43 ± 0.27	0.21 ± 0.12	0.20 ± 0.13	0.22 ± 0.11
Sm		0.01 ± 0.01	0.06 ± 0.09	0.01 ± 0.02	0.01 ± 0.01	0.02 ± 0.02	0.09 ± 0.12	0.05 ± 0.06	0.13 ± 0.15	0.01 ± 0.01	0.01 ± 0.01	0.01 ± 0.01	0.03 ± 0.02	0.02 ± 0.02	0.03 ± 0.03
Zn		12.6 ± 14.7	11.2 ± 9.2	17.9 ± 19.0	20.4 ± 19.6	15.6 ± 18.0	13.0 ± 11.2	12.6 ± 9.6	13.4 ± 12.7	7.28 ± 4.89	7.39 ± 5.32	7.16 ± 4.34	9.44 ± 6.25	8.22 ± 4.87	10.8 ± 7.3

## Supplementary Materials

The following are available online at https://www.mdpi.com/1660-4601/17/15/5447/s1, Figure S1: Spearman correlations between PM and PM components sampled in the studied monitoring stations: “Quebedo” (urban traffic type) and “Mitrena” (industrial type). All elements/ions are displayed in ng·m^−3^ and PM mass concentration is displayed in mg·m^−3^, Figure S2: Contribution of each source to total PM_10_ mass (top) and total PM_2.5_ mass (bottom) sampled in the monitoring stations Quebedo and Mitrena, Table S1: Statistical data (yearly mean [minimum-maximum]) of meteorological variables (relative humidity and temperature, with the number of measurements, n) registered in the two meteorological stations during the monitoring period of 2004–2012, Table S2: Air quality limit and target values established by the European Commission’s Directive 2008/50/EC [51] and air quality guidelines (AQG) defined by the World Health Organization (WHO) [29].
