# Supplementary material for: Long-Term Assessment of Air Quality and Identification of Aerosol Sources at Setúbal, Portugal
Source: Int J Environ Res Public Health. 2020 Jul 28;17(15):5447. doi: 10.3390/ijerph17155447 (PMC7432894; doi:10.3390/ijerph17155447)
Supplement: Supplementary file 1 [file ijerph-17-05447-s001.pdf]

Appendix

Table S1. Statistical data (yearly mean [minimum-maximum]) of meteorological variables (relative humidity and temperature, with the number of measurements, n) registered in the two meteorological stations during the monitoring period of 2004-2012.

	Year								
**Parameter**	**2004**	**2005**	**2006**	**2007**	**2008**	**2009**	**2010**	**2011**	**2012**
Relative Humidity, %	71 [22-94]	69 [20-94]	72 [7-94]	70 [20-94]	72 [22-94]	70 [0-94]	68 [12-97]	68 [14-99]	74 [11-99]
Temperature, ºC	16 [1-37]	16 [0-36]	17 [0-36]	16 [1-36]	16 [1-34]	17 [0-34]	18 [3-39]	17 [4-36]	13 [2-29]
n	8695	8719	8678	8538	8760	5883	6867	8755	8762

Table S2. Air quality limit and target values established by the European Commission's Directive 2008/50/EC [51] and air quality guidelines (AQG) defined by the World Health Organization (WHO) [29].

		EU Air Quality Directive		WHO Guideline (AQG)
**Pollutant**	Averaging Period	Limit value or target concentration	Allowed exceedances per year	Concentration
NO_2	Hourly	200 $\mu g \cdot m^{-3}$	18	200 $\mu g \cdot m^{-3}$
	Annual	40 $\mu g \cdot m^{-3}$		40 $\mu g \cdot m^{-3}$
NOx	Annual	30 $\mu g \cdot m^{-3}$*	-	-
$PM_{2.5}$	Daily	-	-	25 $\mu g \cdot m^{-3}$
	Annual	25 $\mu g \cdot m^{-3}$		10 $\mu g \cdot m^{-3}$
PM_{10}	Daily	50 $\mu g \cdot m^{-3}$	35	50 $\mu g \cdot m^{-3}$
	Annual	40 $\mu g \cdot m^{-3}$	-	20 $\mu g \cdot m^{-3}$
O_3	8 Hours	120 $\mu g \cdot m^{-3}$	25	100 $\mu g \cdot m^{-3}$
SO_2	Hourly	350 $\mu g \cdot m^{-3}$	24	20 $\mu g \cdot m^{-3}$
	Daily	125 $\mu g \cdot m^{-3}$	3	-

* Limit value for protection of vegetation.

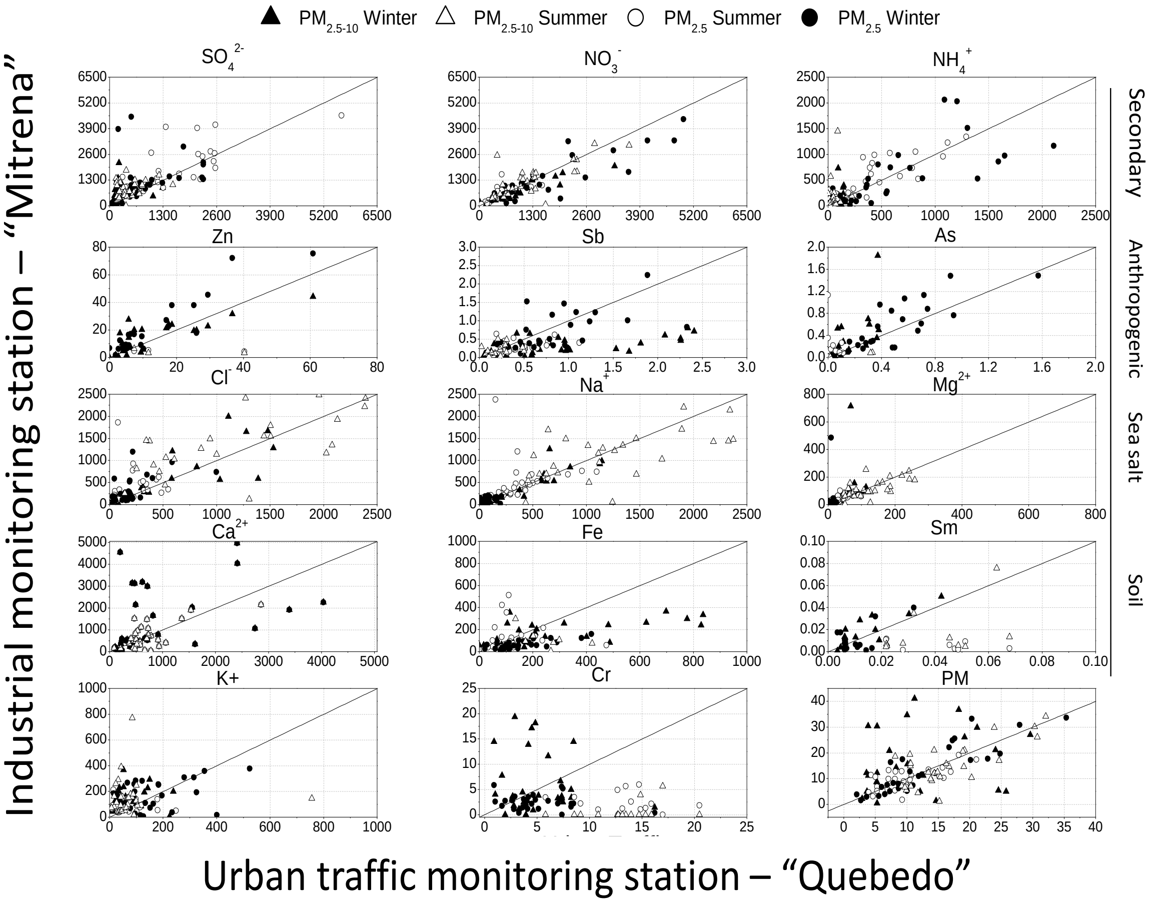

Figure S1. Spearman correlations between PM and PM components sampled in the studied monitoring stations: "Quebedo" (urban traffic type) and "Mitrena" (industrial type). All elements/ions are displayed in ng·m^{-3} and PM mass concentration is displayed in mg·m^{-3}.

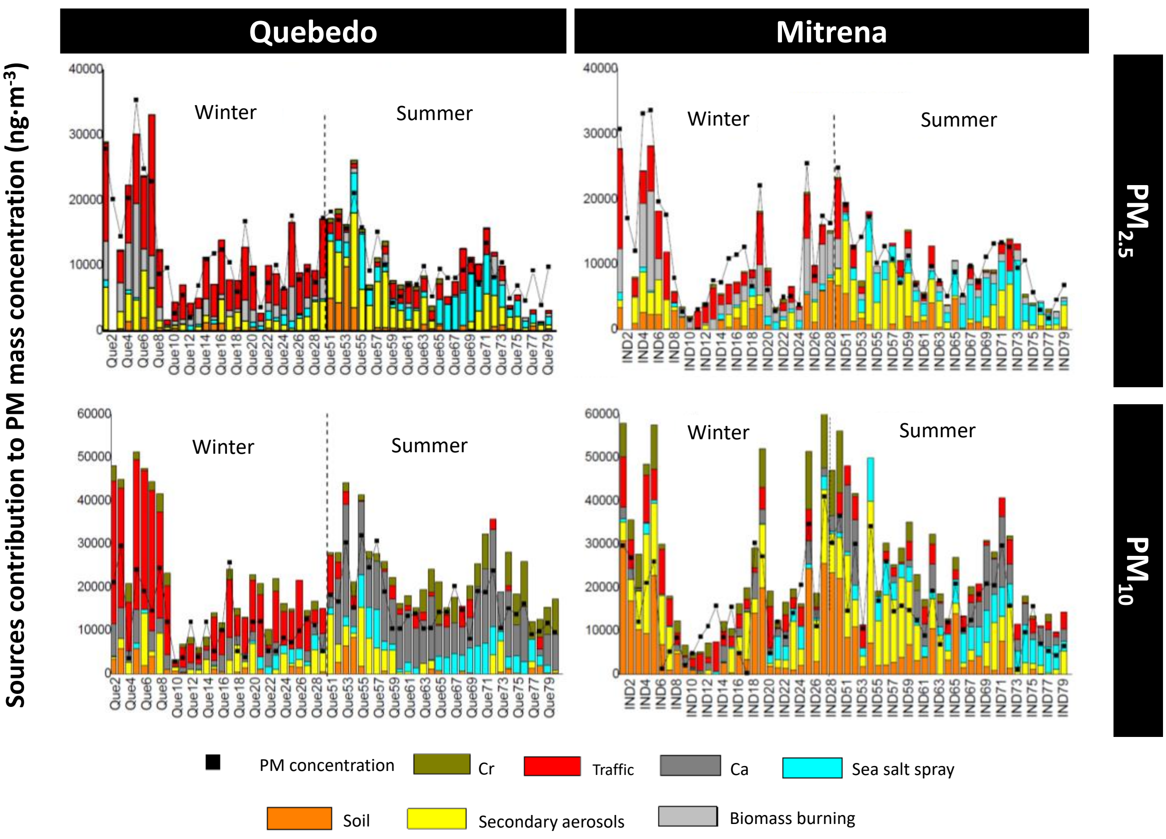

Figure S2. Contribution of each source to total PM_{10} mass (top) and total $PM_{2.5}$ mass (bottom) sampled in the monitoring stations Quebedo and Mitrena.
